# UPLC-DAD/Q-TOF-MS Based Ingredients Identification and Vasorelaxant Effect of Ethanol Extract of Jasmine Flower

**DOI:** 10.1155/2014/707908

**Published:** 2014-05-21

**Authors:** Yongqiang Yin, Xuhui Ying, Hairong Luan, Zhenying Zhao, Jianshi Lou, Deli Wang, Hailin Li, Hong Wu

**Affiliations:** ^1^Department of Pharmacology, Tianjin Medical University, Tianjin 300070, China; ^2^College of Pharmacy, Nankai University, Tianjin 300071, China; ^3^Center of Functional Experiment Teaching and Key Laboratory of Cancer Prevention Research, Mudanjiang Medical University, Mudanjiang, Heilongjiang 157011, China; ^4^Department of Pharmacy, Tianjin Union Medical Center, Tianjin 300121, China

## Abstract

Chinese people commonly make jasmine tea for recreation and health care. Actually, its medicinal value needs more exploration. In this study, vasorelaxant effect of ethanol extract of jasmine flower (EEJ) on isolated rat thoracic aorta rings was investigated and [Ca^2+^] was determined in vascular smooth muscle cells by laser scanning confocal microscope (LSCM). The result of aorta rings showed that EEJ could cause concentration-dependent relaxation of endothelium-intact rings precontracted with phenylephrine or KCl which was attenuated after preincubation of the rings with L-NAME and three different K^+^ channel inhibitors; however, indomethacin and glibenclamide did not affect the vasodilatation of EEJ. In addition, EEJ could inhibit contraction induced by PE on endothelium-denuded rings in Ca^2+^-free medium as well as by accumulation of Ca^2+^ in Ca^2+^-free medium with high K^+^. LSCM also showed that EEJ could lower the elevated level of [Ca^2+^] induced by KCl. These indicate that the vasodilation of EEJ is in part related to causing the release of nitric oxide, activation of K^+^ channels, inhibition of influx of excalcium, and release of calcium from sarcoplasmic reticulum. A total of 20 main ingredients, were identified in EEJ by UPLC-DAD/Q-TOF-MS. The vasodilation activity should be attributed to the high content of flavonoid glycosides and iridoid glycosides found in EEJ.

## 1. Introduction

Jasmine (*Jasminum sambac *(L.)), one of perennial evergreen shrub, is mostly distributed in tropical Asia and temperate regions in Europe and Africa. Their roots, leaves, and flowers have medicinal values [1]. Its flower is widely used in tea industries nowadays and it has been utilized as traditional medicines in early China to treat a variety of diseases such as diarrhea and fever, which was recorded in one of the famous traditional Chinese medical classics called “Compendium of Materia Medica” (Bencao Gangmu, AD 1578). There have few literatures reported on the ingredients in their roots and flowers by column chromatography, gas chromatography (GC), and thin-layer chromatography (TLC) [2–7]; however, there still have no systemic analysis of the ingredients in its flower by liquid chromatography coupled with mass spectrometry (LC-MS) method. Though jasmine has been used as traditional medicine for a long time, there has been rare research about its pharmacological effects mainly on sedative-hypnotic and anesthesia-analgesic action of its root extracts and fewer on cardiovascular pharmacological effects [8, 9], and very few research reported on the vasodilation effect of jasmine flower which mainly focused on the action of causing the release of nitric oxide by EEJ [10]. Our previous study reported vasodilation effect of aqueous extract of jasmine [11]. Continuously, in this study, UPLC-DAD/Q-TOF-MS technology was adopted to identify the main ingredients in the ethanol extract of jasmine flower in order to explore its mechanism of vasorelaxant effect for better clinical use, apart from the action of causing the release of nitric oxide, activating K^+^ channels, and decreasing intracellular calcium level which were investigated using isolated rat thoracic aorta rings and laser scanning confocal microscope (LSCM).

## 2. Materials and Methods

### 2.1. Chemicals and Reagents

LC-MS-grade acetonitrile and HPLC-grade methanol were purchased from Fisher Scientific (Fair Lawn, NJ, USA). Formic acid (98%) was purchased from Acros Organics (Geel, Belgium). N^G^-nitro-L-arginine methyl ester (L-NAME), indomethacin (Indo), 4-aminopyrimide, (4-AP), tetraethylammonium (TEA), glibenclamide (GLi), BaCl_2_, phenylephrine (PE), carbacholine (CCH), EGTA, DMSO, and Fluo-3/AM were purchased from Invitrogen (CA, USA). Water was purified with a Milli-Q system (Millipore, Bedford, USA). Other reagents were of analytical grade.

### 2.2. Preparation of Crude Sample of EEJ

Dried jasmine flower was purchased from Chinese herb market, Anguo, Hebei, China, and authenticated by Dr. Chunfeng Xie of Nanakai University. 50 g jasmine flower was refluxed twice with 80% ethanol (500 mL) for 2 h. The filtered supernatants were combined and condensed under decompression at 30°C. The residue was dissolved in water and extracted with petroleum ether for four times. Water-soluble portion was condensed under decompression to get the crude sample (5.9 g). And then it was prepared in water at concentration of 50 g/L for further use. 1 mL of solution was taken and diluted to 10 mL with water, followed by filtration with a 0.22 *μ*m filter before UPLC-DAD/Q-TOF-MS analysis.

### 2.3. UPLC-DAD/Q-TOF-MS Analysis

EEJ solution was analyzed by a Waters Acquity UPLC chromatographic system (Waters Corp., Milford, USA) coupled with a Waters Q-TOF premier instrument with electrospray ionization system (Waters MS Technologies, Manchester, UK). Chromatographic separation was carried out on Waters Acquity UPLC BEH C18 column (2.1 mm × 100 mm, 1.7 *μ*m) and the temperature of the column oven was maintained at 35°C. The mobile phase system including acetonitrile (A) and water with 0.1% formic acid (B) was performed by gradient elution as follows: 2–20% A from 0–10 min, 20–35% A from 10–15 min, and 35–100% A from 15–17 min. The sample was injected 2 *μ*L for analysis. UV spectra were recorded from 190 to 400 nm. The flow rate was set at 0.4 mL/min. The ESI-MS spectra were acquired in both negative and positive ion modes and the mass range was set from *m*/*z* 100 to 1500. The capillary voltage was set to 2.5 kV for negative and 3.0 kV for positive ion mode. The sample cone voltage was set at 30 V. The desolvation gas flow was set to 600 L/h at a desolvation temperature of 350°C. The cone gas was set to 50 L/h and the source temperature was 110°C. The collision energy of dissociation was set at 35 eV in MS/MS analysis.

### 2.4. Animals

Male Wistar rats (250–300 g) were purchased from the Centre of Laboratory Animals, Harbin Medical University, China. All animals were kept in an animal room with a temperature of 23 ± 2°C, a humidity of 60 ± 5%, and a 12 h dark to light cycle. They had free access to food and water. The animal facilities and protocols were approved by the Institutional Animal Care and Use Committee, Harbin Medical University. All procedures were in accordance with the National Institute of Heath's guidelines regarding the principles of animal care (2004). The experiment animals were housed under the above conditions for a 2-week acclimation period.

### 2.5. Preparation of Rat Aortic Rings

Rat aortic rings were prepared according to [12, 13]. The segment of thoracic aorta was carefully exposed after thoracotomy, dissected free, and quickly placed in chilled (4°C) Kreb's-Henseleit (K-H) solution. Segments were trimmed of adherent adipose and connective tissues and cut into rings in length of 3-4 mm. Endothelium was removed mechanically by gently rubbing the intimal surface of the vessel with fine tipped forceps. The aorta rings were incubated in HV-4 vascular ring perfusion system (Chengdu Taimeng, China) containing 37°C K-H solution continuously bubbled with 95% O_2_ and 5% CO_2_ and were mounted horizontally on two stainless steel hooks. One of the hooks was fixed to the bottom, and the other was connected to a force displacement transducer that was connected to BL-420 biological function experimental system (Chengdu Taimeng, China) and JH-2 muscle tension transducer (Beijing Institute of Space Medico-Engineering). Before starting, 0.5 g resting tension was given to balance for 40 min, and then the resting tension was adjusted to 1.5 g, reequilibrating for 40 min. Buffer was changed every 20 min to prevent the accumulation of metabolites. Maximum contraction amplitude induced by PE (10 *μ*M) was considered as 100%, and the ratio of vascular contraction amplitude to chemicals and max amplitude reflected the changes of vascular tonus.

### 2.6. Effect of EEJ on PE or KCl Precontracted Endothelium-Intact Thoracic Aorta Rings

The vascular rings with endothelium were precontracted with PE (10 *μ*M) or KCl (60 mM) till reaching a contraction plateau and then treated with EEJ (0.125, 0.25, 0.5, 1, and 2 g/L) for 5 min. The effect of each concentration was allowed to reach a steady level before the addition of the next dose.

### 2.7. Influence of L-NAME or Indo on Vasodilatation Effect of EEJ

Aortic rings with endothelium were preincubated with L-NAME (3 mM) or Indo (10 *μ*M) at 37°C for 10 min, followed by precontraction with PE (10 *μ*M) till reaching a contraction plateau, and then treated with EEJ (0.125, 0.25, 0.5, 1, 2 g/L) for 5 min cumulatively.

### 2.8. Influence of Potassium Channel Blockers on Vasodilatation Effect of EEJ

Aortic rings with endothelium were preincubated with potassium channel blockers BaCl_2_ (1 mM), 4-AP (5 mM), TEA (1 mM), and GLi (10 *μ*mM), respectively, at 37°C for 10 min, followed by precontraction with PE (10 *μ*M) till reaching maximum contraction, and then treated with EEJ (0.125, 0.25, 0.5, 1, and 2 g/L) for 5 min cumulatively.

### 2.9. Influence of EEJ on Dose-Response Curve of CaCl_2_

Aortic rings without endothelium were preincubated in Ca^2+^-free K-H solution (containing 100 *μ*M EGTA) for 30 min (changing K-H buffer at 10 min intervals), then add 60 mM KCl for 10 min, followed by addition of five different concentrations of CaCl_2_ (0.5, 1, 2, 4, and 8 mM) for 10 min cumulatively. The EEJ group was incubated with EEJ (1 g/L) for 30 min before adding CaCl_2_.

### 2.10. Influence of EEJ on PE Induced Vasoconstriction in Ca^2+^-Free K-H Solution

Aortic rings without endothelium were preincubated in Ca^2+^-free K-H solution (containing 100 *μ*mol/L EGTA) for 30 min and then precontracted with PE (10 *μ*M) for 10 min, recording max contraction amplitude and calculating tension difference. After several washings and reequilibration, the aortic rings were incubated with Ca^2+^-free K-H solution for 30 min, followed by addition of EEJ (1 g/L) for 10 min, and then treated with PE (10 *μ*M) for 10 min, recording max contraction amplitude and calculating tension difference. Compare the two tension differences to investigate the effect of EEJ on PE-induced vasoconstriction in Ca^2+^-free environment.

### 2.11. Acute Isolation of Rat Thoracic Aortic Smooth Muscle Cells and Detection Intracellular Calcium with Laser Confocal

Acute isolation of rat thoracic aortic smooth muscle was performed as described [14]. Cells were loaded with Fluo-3/AM (20 *μ*M), incubated in 37°C water bath for 45 min, removed the loading buffer, and washed with normal Hank's. Loaded cells were treated with 1 g/L of EEJ, 2 g/L of EEJ, and 10 *μ*M of verapamil for 10 min, respectively, and then adding KCl (30 mM). Confocal scanned real time intracellular Ca^2+^ concentration ([Ca^2+^]_*i*_) was performed at 488/525 nm (Ex/Em) with FV-300 laser scanning confocal microscope (Olympus, Japan). The data are presented as a ratio of *F*_max⁡_/*F*_0_.

### 2.12. Statistical Analysis

Data were presented as mean ± SD. The significance in mean values was analyzed by *t*-test for 2 groups and by analysis of variance (ANOVA) with least squares difference post-hoc test for more than 2 groups. A *P* value of less than 0.05 was regarded as a statistically significant difference.

## 3. Results

### 3.1. Identification Result of EEJ by UPLC-DAD/Q-TOF-MS

**Figure 1:**
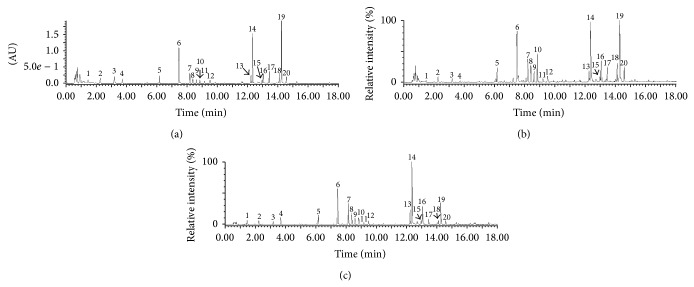
Chromatograms of EEJ analyzed by UPLC-DAD/Q-TOF-MS. (a) UV chromatogram of EEJ (scanned from 190 nm to 400 nm). (b) TIC chromatogram of EEJ in positive ion mode. (c) TIC chromatogram of EEJ in negative ion mode. Peak numbers are consistent with those shown in Table 1.

The typical UV chromatogram (full wavelength scan chromatogram from 200 to 400 nm) and total ion current (TIC) chromatograms (positive ion mode and negative ion mode) of EEJ are shown in Figure 1. The accurate mass ions obtained from positive and negative TIC chromatograms and the fragment ions obtained from MS/MS model are summarized in Table 1. In total, 20 compounds in EEJ were tentatively identified, most of which belonged to flavonoid glycoside, iridoid glycosides, and quercetin-3-O-(2,6-*α*-L-rhamnopyranosyl-*β*-D-glucopyranoside) (peak 6), molihuaside A (peak 14), and sambacoside A (peak 19) with relatively high content.

### 3.2. Effect of EEJ on PE or KCl Precontracted Endothelium-Intact Thoracic Aorta Rings

**Figure 2:**
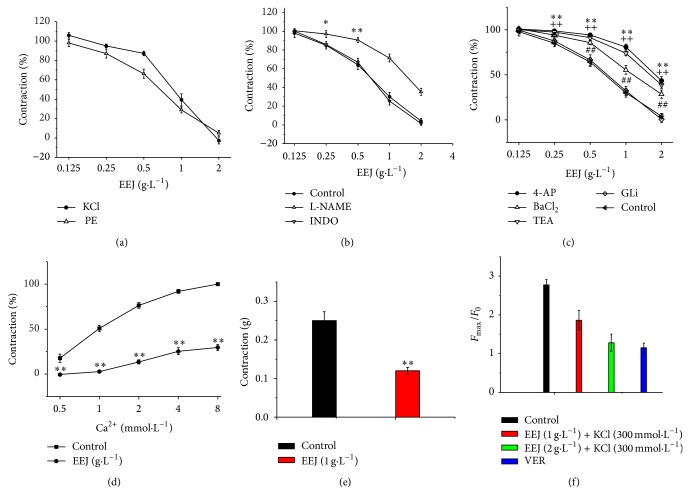
Vasorelaxant effect of EEJ. Data are expressed as mean ± SD (*n* = 8). (a) Cumulative concentration response for EEJ (0.125, 0.25, 0.5, 1, and 2 g/L) on PE (10 *μ*M) or KCl (60 mM) precontracted endothelium-intact thoracic aorta rings. Maximum contraction amplitude induced by PE (10 *μ*M) is considered as 100%. (b) Effects of L-NAME (3 mM) and Indo (10 *μ*M) on EEJ induced relaxation in the endothelium-intact aorta rings precontracted with PE (10 *μ*M). ^*^ *P* < 0.05,^**^ *P* < 0.01 compared with control group. (c) Effects of 4-AP (5 mM), BaCl_2_ (1 mM), TEA (1 mM), and GLi (10 *μ*M) on EEJ induced relaxation in the endothelium-intact aorta rings precontracted with PE (10 *μ*M). ^**^ *P* < 0.01 compared 4-AP treating group with control group;^++^ *P* < 0.01 compared TEA treating group with control group;^##^ *P* < 0.01 compared BaCl_2_ treating group with control group. (d) Effects of EEJ (1 g/L) on the cumulative concentration response for CaCl_2_ (0.5 mM–8 mM) in high K^+^ (60 mM)-Ca^2+^-free depolarizing solution. ^**^ *P* < 0.01 compared with control group. (e) Effect of EEJ (1 g/L) on the PE (10 *μ*M) precontracted endothelium-denuded aortic rings in the Ca^2+^-free K-H solution. ^**^ *P* < 0.01 compared with the Ca^2+^-containing group. (f) Influence of EEJ (1 g/L, 2 g/L) on the increase of [Ca^2+^]_*i*_ in ASMCs induced by KCl (30 mM). Verapamil (VER, 10 *μ*M) was applied as the positive inhibition control group. ^**^ *P* < 0.01 EEJ (1 g/L) treating group compared with the control group; ^##^ *P* < 0.01 EEJ (2 g/L) treating group compared with the control group; ^++^ *P* < 0.01 verapamil treating group compared with the control group.

As shown in Figure 2(a), EEJ caused vasodilation of the endothelium-intact thoracic aorta ring preconstricted with PE in a concentration-dependent manner. The max vasodilatation amplitude was 103 ± 3.6% and 96 ± 3.1% for KCl and PE precontraction, respectively.

### 3.3. Influence of L-NAME or Indo on Vasodilatation Effect of EEJ

As shown in Figure 2(b), there has no significant difference (*P* > 0.05) on the vasodilation effect of EEJ on PE precontracted aortic rings with endothelium in presence or absence of Indo, a cyclooxygenase inhibitor. However, the vasodilation effect of EEJ on PE precontracted aortic rings with endothelium was significantly decreased after preincubation of the aortic rings with L-NAME, a NO synthase inhibitor. The max relaxation amplitude was 65.3 ± 3.5%, a significant difference (*P* < 0.05) compared to the control group.

### 3.4. Influence of Potassium Channel Blockers on Vasodilatation Effect of EEJ

Figure 2(c) shows that voltage-sensitive K^+^ channels (K_v_) inhibitor 4-AP, inwardly rectifying K^+^ channels (K_IR_) inhibitor BaCl_2_, and Ca^2+^-activated K^+^ channel (K_Ca_) inhibitor TEA significantly attenuate vasodilation effect of EEJ on PE precontracted aortic rings, with the max relaxation amplitude of 57 ± 4.5%, 78 ± 4.6%, and 61 ± 3.6%, respectively, a significant difference compared with the control group (*P* < 0.01). Nevertheless, there was no significant decrease in the percentage maximum relaxation response of PE precontracted aortic rings with preincubation of GLi, a K_ATP_ nonspecific inhibitor, when compared with PE precontracted aortic rings without preincubation of GLi.

### 3.5. Influence of EEJ on Dose-Response Curve of CaCl_2_

Figure 2(d) shows that the dose-response curve of CaCl_2_ was shifted to right and the contraction was significantly (*P* < 0.01) decreased when the aorta rings were pretreated with EEJ (1 g/L) in Ca^2+^-free K-H solution (containing 100 *μ*M EGTA).

### 3.6. Influence of EEJ on PE Induced Vasoconstriction in Ca^2+^-Free K-H Solution

As shown in Figure 2(e), EEJ significantly inhibited PE induced vasoconstriction in Ca^2+^-free K-H solution, and the max tension was reduced by 0.13 ± 0.014 g, a significant difference compared with the control group (*P* < 0.01).

### 3.7. Influence of EEJ on Cytoplasm [Ca^2+^]_*i*_ of ASMC

After loaded with Fluo-3/AM, ASMCs were scanned with confocal. As shown in Figure 2(f), EEJ inhibited KCl-induced [Ca^2+^]_*i*_ increase in a dose-dependent manner. The *F*_max⁡_/*F*_0_ of [Ca^2+^]_*i*_ was at 1.86 ± 0.25 when 1 g/L of EEJ was added, while the *F*_max⁡_/*F*_0_ of [Ca^2+^]_*i*_ was decreased to 1.28 ± 0.22 when 2 g/L of EEJ was added; both showed significant difference (*P* < 0.01) compared with the control group whose *F*_max⁡_/*F*_0_ of [Ca^2+^]_*i*_ was at 2.77 ± 0.14.

## 4. Discussions

Jasmine flower is widely used as a tea in the world, especially in East Asia, and it has been traditionally used in ancient China as a medicine treating diarrhea and conjunctivitis. Nevertheless, the ingredients and pharmacological activities of jasmine flower have been rarely reported, especially on its vasodilation effect.

In UPLC-DAD/Q-TOF-MS analysis, the chromatographic conditions for analysis of EEJ were optimized by comparing different mobile phase systems with different gradient elution programs (methanol/water and acetonitrile/water), different water phase additives with different concentrations (formic acid and acetic acid with a concentration of 0.05%, 0.1%, and 0.5%, resp.), and different column temperatures (30, 35, and 40°C). Finally, a rapid analytical method with good separation for most of compounds in EEJ was established.

The accurate mass ions, fragment ions and maximum UV absorptions of peaks 1, 2, and 4 were consistent with previous report [15]. Hence, they were identified as guanosine, phenylalanine, and tryptophan, respectively. Peak 3 showed a positively charged molecular ion [M+H]^+^ at *m*/*z* 357 and negatively charged molecular ion [M−H]^−^ at *m*/*z* 355 indicating a MW of 356. It gave major fragment ion by loss of *m*/*z* 162 (glucose-H_2_O) in both positive and negative ion MS/MS analysis. Additionally, it exhibited a characteristic UV absorption at 320 nm. So it was presumed as* trans*-p-feruloyl-*β*-D-glucopyranoside which had been identified in a medicinal flower [16]. Peak 10 showed a MW of 590 Da deduced from MS results and was tentatively identified as* trans*-p-ferulylalcohol-4-O-(2-glucopyranosyl-6-(2-methyl-3-hydroxypropionyl)) glucopyranoside (FGG) based on the spectra data of* trans*-p-ferulylalcohol-4-O-(6-(2-methyl-3-hydroxypropionyl) glucopyranoside (FG) which showed a MW of 428 Da reported in [17]. Peak 10 had similar characteristic UV absorption at 250 and 340 nm with FG predicting that peak 10 probably had the same structure skeleton with FG. It could form fragment ions at *m*/*z* 429 in positive ion model and *m*/*z* 427 in negative ion model by loss of 162 Da corresponding to glucopyranose which means that peak 10 has one glucopyranosyl group more than FG. In addition, peak 10 could also form fragment ions at *m*/*z* 411 in positive ion model by losses of 162 Da (glucose-H_2_O) and cleave off a H_2_O at 2-methyl-3-hydroxypropionyl side chain. Similarly, peak 5 was identified as benzyl 6-O-(*β*-D-xylopyranosyl)-*β*-D-glucopyranoside which could form a fragment of benzyl-O-*β*-D-glucopyranoside, and a compound had been earlier reported in a medicinal flower [16] and food [18], by loss of one xylopyranosyl group.

Peaks 6, 7, 8, 9, 11, and 12 were all flavonoid glycoside taking quercetin or kaempferol as aglycone. They were identified based on their UV and mass spectral data compared to those in the literature [16, 18]. Normally, these compounds could be losses of glucopyranosyl or/and rhamnopyranosyl groups in the MS/MS analysis. For some compounds with quercetin as aglycone, quercetin-free radical fragment was formed at *m*/*z* 300 in negative ion MS/MS analysis.

Peaks 13, 14, 15, 16, 17, 18, 19, and 20 that were all iridoid glycosides showed same maximum UV absorptions at 191 and 237 nm. They were confirmed by the mass spectral data and contents of them reported earlier in jasmine [19, 20]. Peaks 13, 14, 15, and 16 were isomers, and they could form fragments at *m*/*z* 797 and 617 in positive mode by losses of one and two glucopyranoses, respectively. And the fragment of *m*/*z* 393 was formed by further loss of one molecular iridoid aglycone. They gave the fragments of *m*/*z* 813 and 589 in negative mode by losses of glucopyranosyl and one molecular iridoid glycoside, respectively. Similarly, peaks 17, 18, 19, and 20 showed the main fragments in positive mode by losses of one or more glucopyranoses or iridoid glycosides while giving the main fragments in negative mode by losses of one glucopyranosyl and one molecular iridoid glycoside.

Endothelium plays vital roles in aortic ring contraction/relaxation. Vasodilators, such as prostaglandin I_2_ (PGI_2_) and endothelial derived relaxing factor (EDRF) or nitric oxide (NO), were synthesized and released by vascular endothelium [21, 22]. NO is a potent vasodilator synthesized in the endothelium by NO synthase, and activating M_3_ cholinergic receptors on endothelial cells can also lead to the release of NO [23]. NO causes vascular relaxation by stimulating guanylate cyclase, increasing intracellular cGMP, and decreasing Ca^2+^. PGI_2_ is synthesized by prostacyclin synthase in endothelial cells [24]. As shown in Figure 2(a), EEJ could antagonize PE or KCl-induced vasoconstriction in a concentration-dependent manner. But this effect could be significantly attenuated by L-NAME, a NO synthase inhibitor, as shown in Figure 2(b), which is consistent with a previous report [10]; however, indole, a prostacyclin synthase inhibitor, did not influence on the vasodilatation of EEJ. It indicates that the vasodilatation effect of EEJ might be related to M_3_ receptor or L-arginine/NO pathway but not to PGI_2_ pathway.

Ion channels, such as K^+^ channel and Ca^2+^ channel, also participate in vascular smooth muscle contraction/relaxation. K^+^ channels play important roles in regulating of VSM contraction and vascular tonus. Activating of K^+^ channels in VSM leads to vasodilation by cellular membrane hyperpolarization and inhibiting the influx of extracellular calcium [25]. There are four types of K^+^ channels in VSM: ATP-sensitive K^+^ channels (K_ATP_), Ca^2+^ activated K^+^ channels (K_ca_), voltage-sensitive K^+^ channels (Kv), and inward rectifier-type K^+^ channels (K_IR_). TEA, 4-AP, and BaCl_2_ could inhibit Kca, Kv, and K_IR_, respectively [26]. As shown in Figure 2(c), the vasodilation effect of EEJ was partially inhibited when the vascular rings were preincubated with TEA, 4-AP, or BaCl_2_ before treating with EEJ, indicating that the vasodilatation of EEJ is associated with several K^+^ channels. K_Ca_, K_v_, and K_IR_ might participate in EEJ vasodilatation, but not K_ATP_.

There are two types of Ca^2+^ channels in VSM cell membrane [27], including voltage dependent calcium channel (VDCC) and receptor operated calcium channel (ROCC). VDCC is regulated voltage-dependently by membrane potential. ROCC is coupled with membrane receptors. Specific ROCC receptor agonists, such as PE, can bind the receptor and activate ROCC, which prompt the release of intracellular Ca^2+^ from sarcoplasmic reticulum (SR) and influx of extracellular Ca^2+^, leading to vasoconstriction [28]. PE can also activate *α*1 receptors in vessels, promoting the release of intracellular Ca^2+^ from SR. Both pathways lead to the elevation of intracellular Ca^2+^ and contraction of VSM regulated by calmodulin. High level of extracellular K^+^ predisposes that the VDCC is activated by depolarizing membrane, leading to influx of extracellular Ca^2+^ and contraction of vessel [29]. As seen from Figure 2(d), EEJ attenuated the vasoconstriction to cumulative calcium in high potassium medium without Ca^2+^, suggesting that the vasodilatation effect of EEJ is related to inhibiting the influx of extracellular Ca^2+^ mediated by VDCC. In Ca^2+^-free buffer, PE could cause vasoconstriction by inducing the release of SR Ca^2+^, which was significantly attenuated by EEJ, as shown in Figure 2(e). The results indicated that inhibiting release of Ca^2+^ from SR was involved in vasodilatation of EEJ, as for mediated by ROCC or *α*_1_ receptor pathway would be under further investigation [30, 31].

The increased concentration of intracellular Ca^2+^ directly leads to smooth muscle contraction. EEJ generally relaxed aorta rings precontracted with PE or KCl, providing evidence that EEJ affected intracellular Ca^2+^. The confocal data in Figure 2(f) showed that the elevation of intracellular Ca^2+^ induced by KCl could be assuaged by EEJ in a concentration-dependent manner, indicating that vasodilatation effect of EEJ is related to decrease of intracellular Ca^2+^. This result was consistent with vascular ring testing.

To our knowledge, how chemicals affect aorta rings mainly lies in four mechanisms, endothelium dependent, K^+^ channel related, Ca^2+^ channel, and receptor dependent. In this study, we found that EEJ mechanisms of vasorelaxant effect at least partly lie in causing the release of nitric oxide, activating K^+^ channels, and decreasing intracellular calcium level. It is known that, the endothelium integrity of hypertension patients varies. In our study, we found that, besides NO release pathway, EEJ also had effects on K^+^ channel and Ca^2+^ channel, indicating that EEJ exhibits its vasorelaxant role in multimechanism, which is of clinical significance.

According to our LC-MS result, the main ingredients in EEJ are flavonoid glycosides and iridoid glycosides. It has been reported that the vasorelaxant property of most plant extracts is related to flavonoids [32–36]. In addition, there have several reports on the vasorelaxant effects of iridoid glycosides as well [37]. Hence, the vasodilation activity of EEJ should be attributed to flavonoid glycosides and iridoid glycosides found in EEJ, especially quercetin-3-O-(2,6-*α*-L-dirhamnopyranosyl-*β*-D-glucopyranoside), molihuaside A, and sambacoside A which are the relatively high content of flavonoid glycosides and iridoid glycosides identified in EEJ. This study provides useful information for better application of jasmine flower in traditional medicines and tea industries. Nevertheless, further studies are necessary to clearly elucidate the specific ingredients which responsible for such effects and further investigate the mechanism of vasodilation effects of EEJ on molecular level, as well as in vivo study being potentially affected with neural or humor factors.

## 5. Conclusion

In this study, we have a preliminary understanding of the vasodilation effects and potential mechanism of EEJ and find that EEJ has a concentration-dependent relaxation in rat aorta rings by stimulating nitric oxide release, activating multiple potassium channels on VSM, inhibiting influx of extracellular Ca^2+^, and preventing the release of Ca^2+^ from SR. The vasodilation activity of EEJ should be attributed to the high content of flavonoid glycosides and iridoid glycosides found in EEJ.

## Figures and Tables

**Table 1:** Identification of the ingredients in EEJ by UPLC-DAD/Q-TOF-MS.

Number	*t*_*R*_ (min)	Positive ion	Negative ion	UV (*λ*max)	Identification	Exact mass
		[M + H]^+^ (MS/MS)	[M − H]^−^ (MS/MS)			
1	1.44	284.0995 (152)	282.0846 (150)	205; 258	Guanosine	283.0917
2	2.23	166.0829 (149; 120)	164.0661 (147)	205; 258	Phenylalanine	165.0790
3	3.17	357.1244 (193)	355.1105 (191)	230; 321	*trans * -p-Feruloyl-*β*-D-glucopyranoside	356.1107
4	3.63	205.0961 (188; 132)	203.0784 (159; 130)	219; 278	Tryptophan	204.0899
5	6.17	403.1629 (271)	401.1412 (269; 161)	203; 255	Benzyl 6-O-(*β*-D-xylopyranosyl)-*β*-D-glucopyranoside	402.1526
6	7.45	757.2231 (611; 465; 303)	755.2079 (301; 300)	254; 353	Quercetin-3-O-(2,6-*α*-L-dirhamnopyranosyl-*β*-D-glucopyranoside)	756.2113
7	8.16	741.2261 (595; 449; 287)	739.2090 (575; 285; 284)	230; 347	Kaempferol-3-O-(2,6-*α*-L-dirhamnopyranosyl-*β*-D-glucopyranoside)	740.2164
8	8.37	611.1608 (465; 303)	609.1449 (301; 300)	255; 353	Rutin	610.1534
9	8.57	611.1613 (465; 303)	609.1456 (301; 300)	255; 352	Quercetin-3-O-neohesperidoside	610.1534
10	8.87	591.2371 (429; 411)	589.2212 (427)	249; 339	*trans*-p-Ferulylalcohol-4-O-(2-glucopyranosyl-6-(2-methyl-3-hydroxypropionyl)) glucopyranoside	590.2211
11	9.19	595.1647 (449; 287)	593.1499 (427; 285)	230; 348	Kaempferol-3-O-*β*-rutinoside	594.1585
12	9.52	449.1030 (287)	447.0866 (285)	230; 348	Kaempferol-3-O-*β*-D-glucopyranoside	448.1006
13	12.21	977.3852 (797; 617; 393)	975.3782 (813; 589)	191; 237	Molihuaside E	976.3788
14	12.35	977.3931 (797; 617; 393)	975.3814 (813; 589)	191; 237	Molihuaside A	976.3788
15	12.93	977.3994 (797; 617; 393)	975.3796 (813; 589)	191; 237	Molihuaside D	976.3788
16	13.03	977.3879 (797; 617; 393)	975.3778 (813; 589)	191; 237	Molihuaside C	976.3788
17	13.42	1363.5467 (1183; 1003; 823; 375)	1361.5055 (1199; 813)	191; 237	Molihuaside B	1362.5000
18	14.08	1363.5112 (1183; 1003; 823; 375)	1361.5082 (1199; 813)	191; 237	Sambacoside E	1362.5000
19	14.23	1363.5226 (1183; 1003; 823; 375)	1361.5073 (1199; 813)	191; 237	Sambacoside A	1362.5000
20	14.55	1363.5189 (1183; 1003; 823; 375)	1361.5036 (1199; 813)	191; 237	Sambacoside F	1362.5000

## References

[1] Liu H., Ni W., Yuan M., Chen C. (2004). The chemical constituents of *Jasminum sambac*. *Acta Botanica Yunnanica*.

[2] Somanadhan B., Smitt U. W., George V. (1998). Angiotensin Converting Enzyme (ACE) inhibitors from *Jasminum azoricum* and *Jasminum grandiflorum*. *Planta Medica*.

[3] Inagaki J., Watanabe N., Moon J. H. (1995). Glycosidic aroma precursors of 2-phenylethyl and benzyl alcohols from *Jasminum sambac* flowers. *Bioscience, Biotechnology, and Biochemistry*.

[4] Zhang Z.-F., Bian B.-L., Yang J., Tian X.-F. (2004). Studies on chemical constitutents in roots of *Jasminum sambac*. *China Journal of Chinese Materia Medica*.

[5] Edris A. E., Chizzola R., Franz C. (2008). Isolation and characterization of the volatile aroma compounds from the concrete headspace and the absolute of *Jasminum sambac* (L.) Ait. (Oleaceae) flowers grown in Egypt. *European Food Research and Technology*.

[6] Zeng L. H., Hu M., Yan Y. M., Lu Q., Cheng Y. X. (2012). Compounds from the roots of *Jasminum sambac*. *Journal of Asian Natural Products Research*.

[7] Pragadheesh V. P. P. S., Yadav A., Chanotiya C. S., Rout P. K., Uniyal G. C. (2011). Monitoring the emission of volatile organic compounds from flowers of *Jasminum sambac* using solid-phase micro-extraction fibers and gas chromatography with mass spectrometry detection. *Natural Product Communications*.

[8] Rath C., Devi S., Dash S., Mishra R. (2008). Antibacterial potential assessment of Jasmine essential oil against *E. coli*. *Indian Journal of Pharmaceutical Sciences*.

[9] Chan P. T., Fong W. P., Cheung Y. L., Huang Y., Ho W. K. K., Chen Z.-Y. (1999). Jasmine green tea epicatechins are hypolipidemic in hamsters (*Mesocricetus auratus*) fed a high fat diet. *Journal of Nutrition*.

[10] Kunhachan P., Banchonglikitkul C., Kajsongkram T., Khayungarnnawee A., Leelamanit W. (2012). Chemical composition, toxicity and vasodilatation effect of the flowers extract of *Jasminum sambac* (L.) Ait. “g. Duke of Tuscany”. *Evidence-based Complementary and Alternative Medicine*.

[11] Luan H.-R., Yin J.-J., Mo W.-H., Zhang B.-N., Pang X.-P., Hou Y.-L. (2010). Vasodilation effect of aqueous extract of jasmine on rat thoracic aorta and its related mechanism. *Chinese Pharmaceutical Journal*.

[12] Chen Z.-Y., Su Y.-L., Lau C.-W., Law W.-I., Huang Y. (1999). Endothelium-dependent contraction and direct relaxation induced by baicalein in rat mesenteric artery. *European Journal of Pharmacology*.

[13] Ko W.-H., Yao X.-Q., Lau C.-W. (2000). Vasorelaxant and antiproliferative effects of berberine. *European Journal of Pharmacology*.

[14] Honda H., Unemoto T., Kogo H. (1999). Different mechanisms for testosterone-induced relaxation of aorta between normotensive and spontaneously hypertensive rats. *Hypertension*.

[15] Ying X. H., Ma J. F., Liang Q. L., Wang Y. M., Bai G., Luo G. A. (2013). Identification and analysis of the constituents in an aqueous extract of tricholoma matsutake by HPLC coupled with diode array detection/electrospray ionization mass spectrometry. *Journal of Food Science*.

[16] Yoshikawa M., Sugimoto S., Nakamura S., Matsuda H. (2008). Medicinal flowers. XXII structures of chakasaponins V and VI, chakanoside I, and chakaflavonoside a from flower buds of Chinese tea plant (*Camellia sinensis*). *Chemical and Pharmaceutical Bulletin*.

[17] Materska M., Piacente S., Stochmal A., Pizza C., Oleszekc W., Perucka I. (2003). Isolation and structure elucidation of flavonoid and phenolic acid glycosides from pericarp of hot pepper fruit *Capsicum annuum L*. *Phytochemistry*.

[18] Schwarz B., Hofmann T. (2007). Sensory-guided decomposition of red currant juice (*Ribes rubrum*) and structure determination of key astringent compounds. *Journal of Agricultural and Food Chemistry*.

[19] Tanahashi T., Nagakura N., Inoue K., Inouye H. (1988). Sambacosides a, e and f, novel tetrameric iridoid glucosides from *Jasminum sambac*. *Tetrahedron Letters*.

[20] Zhang Z. Y.-J., Liu L. Y.-Q., Pu P. X.-Y., Yang Y. C.-R. (1995). Iridoidal glycosides from *Jasminum sambac*. *Phytochemistry*.

[21] Rosado E., Rodriguez-Vilarrupla A., Gracia-Sancho J., Monclus M., Bosch J., Garcia-Pagan J. C. (2012). Interaction between NO and COX pathways modulating hepatic endothelial cells from control and cirrhotic rats. *Journal of Cellular and Molecular Medicine*.

[22] Pignone A., Del Rosso A., Brosnihan K. B. (2007). Reduced circulating levels of angiotensin-(1-7) in systemic sclerosis: a new pathway in the dysregulation of endothelial-dependent vascular tone control. *Annals of the Rheumatic Diseases*.

[23] Yang B. (2003). *Pharmacology*.

[24] Yao T. (2003). *Physiology*.

[25] Seino S., Miki T. (2003). Physiological and pathophysiological roles of ATP-sensitive K^+^ channels. *Progress in Biophysics & Molecular Biology*.

[26] Jackson W. F. (2000). Ion channels and vascular tone. *Hypertension*.

[27] Paoletti R., Govoni S. (1987). Classification of calcium antagonists: proposal of the WHO Committee. *Pharmacological Research Communications*.

[28] Achike F. I., Mohamad R., Mustafa M. R., Rampal R. M. (1999). Acidosis-induced vasodilation in the rat aorta: the role of voltage- and receptor-operated calcium channels. *Medical Science Research*.

[29] Rembold C. M. (1992). Regulation of contraction and relaxation in arterial smooth muscle. *Hypertension*.

[30] He J.-Y., Zhang W., He L.-C., Cao Y.-X. (2007). Imperatorin induces vasodilatation possibly via inhibiting voltage dependent calcium channel and receptor-mediated Ca^2+^ influx and release. *European Journal of Pharmacology*.

[31] Fu X.-C., Wang M.-W., Li S.-P., Zhang Y., Wang H.-L. (2005). Vasodilatation produced by orientin and its mechanism study. *Biological & Pharmaceutical Bulletin*.

[32] Heiss C., Keen C. L., Kelm M. (2010). Flavanols and cardiovascular disease prevention. *European Heart Journal*.

[33] Pérez-Vizcaíno F., Ibarra M., Cogolludo A. L. (2002). Endothelium-independent vasodilator effects of the flavonoid quercetin and its methylated metabolites in rat conductance and resistance arteries. *Journal of Pharmacology and Experimental Therapeutics*.

[34] Fitzpatrick D. F., Hirschfield S. L., Ricci T., Jantzen P., Coffey R. G. (1995). Endothelium-dependent vasorelaxation caused by various plant extracts. *Journal of Cardiovascular Pharmacology*.

[35] Chen C. K., Pace-Asciak C. R. (1996). Vasorelaxing activity of resveratrol and quercetin in isolated rat aorta. *General Pharmacology*.

[36] Woodman O. L., Chan E. C. H. (2004). Vascular and anti-oxidant actions of flavonols and flavones. *Clinical and Experimental Pharmacology and Physiology*.

[37] Iizuka T., Sakai H., Moriyama H., Suto N., Nagai M., Bagchi D. (2009). Vasorelaxant effects of forsythide isolated from the leaves of *Forsythia viridissima* on NE-induced aortal contraction. *Phytomedicine*.

